# Supplementary material for: Cancer associated fibroblasts serve as an ovarian cancer stem cell niche through noncanonical Wnt5a signaling
Source: NPJ Precis Oncol. 2024 Jan 8;8:7. doi: 10.1038/s41698-023-00495-5 (PMC10774407; doi:10.1038/s41698-023-00495-5)
Supplement: Supplementary file 2 — Supplementary Figs. 1–12 [file 41698_2023_495_MOESM2_ESM.pdf]

A

Fibroblast Score Gene List		
COL1A1	COL3A1	COL6A1
COL6A2	GREM1	PARM1
DCN	TAGLN	

B

AOCS microarray probe list			
1555724_s_at	201852_x_at	201893_x_at	205547_s_at
202311_s_at	209156_s_at	209335_at	211813_x_at
211896_s_at	212091_s_at	212937_s_at	212938_at
212940_at	213290_at	213661_at	217430_x_at
218468_s_at	218469_at		

Supplementary Figure 1:
A: The Cancer Genome Atlas (TCGA) ovarian cancer data contain both clinical and gene expression profiles from patient samples. Microenvironment Cell Populations-counter (MCP-counter, version 1.2.0) was applied to deconvolve fibroblasts in TCGA dataset. The table lists the genes used by MCP-counter to determine the fibroblast score.
B: The Australian Ovarian Cancer Study (AOCS) dataset (GSE9891) profiled gene expression of 285 ovarian patient samples, segregated into chemo-resistant and chemo-sensitive. The table lists the genes used by MCP-counter (version 1.2.0) to determine the fibroblast score in the microarray data set.

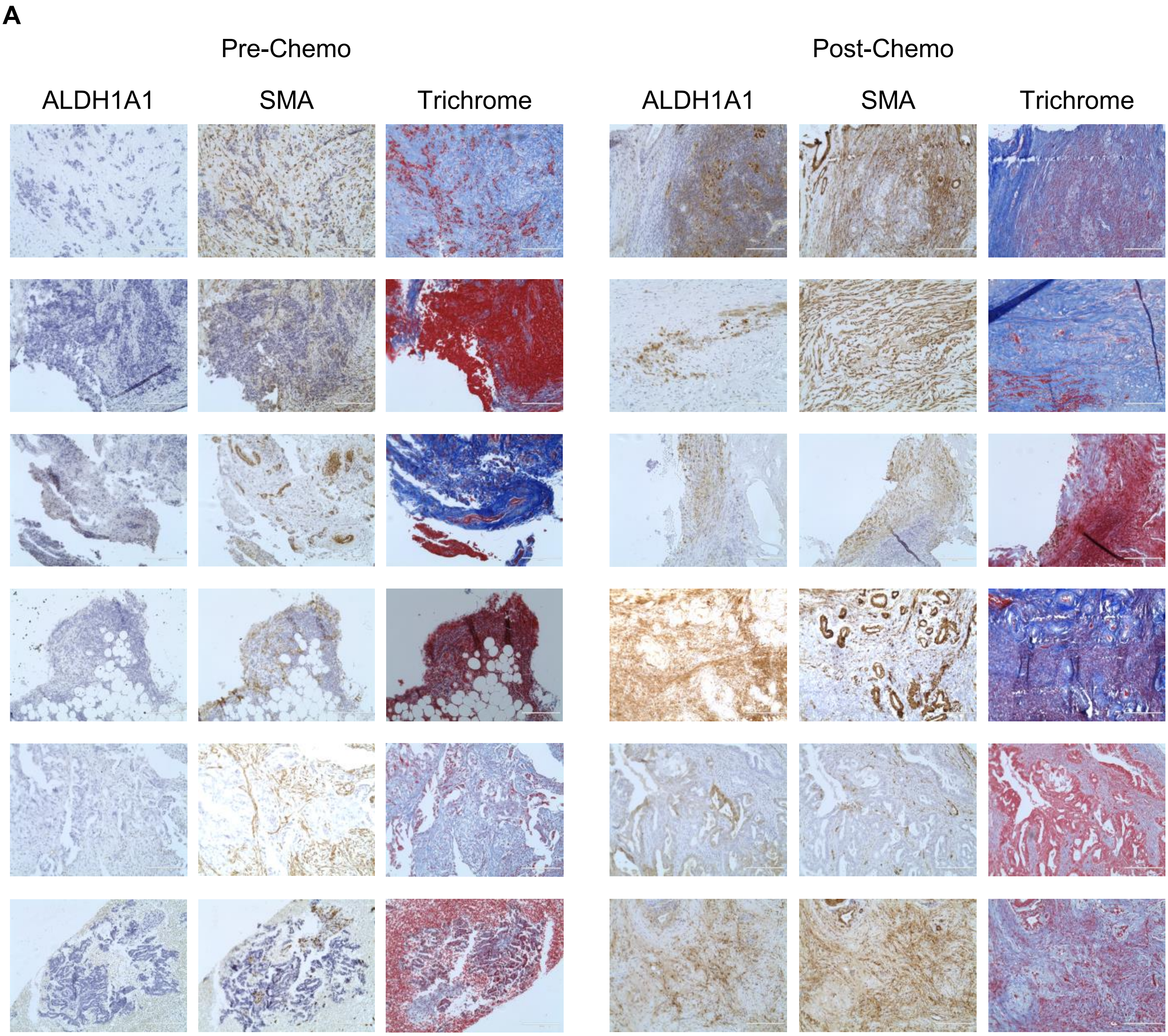

Supplementary Figure 2:
A: Immunohistochemical staining for ALDH1A1 (OCSC marker), αSMA (CAF marker) and Masson's trichrome in 6 additional HGSOC patient omental metastasis pre- and post-chemotherapy (matched). (Scale bar: 200μm)

Supplementary Figure 3

A

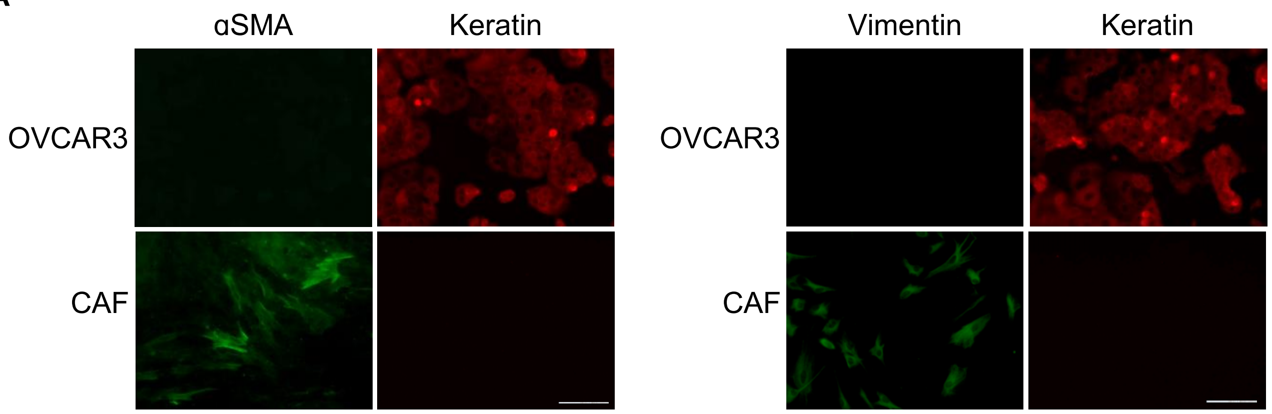

B

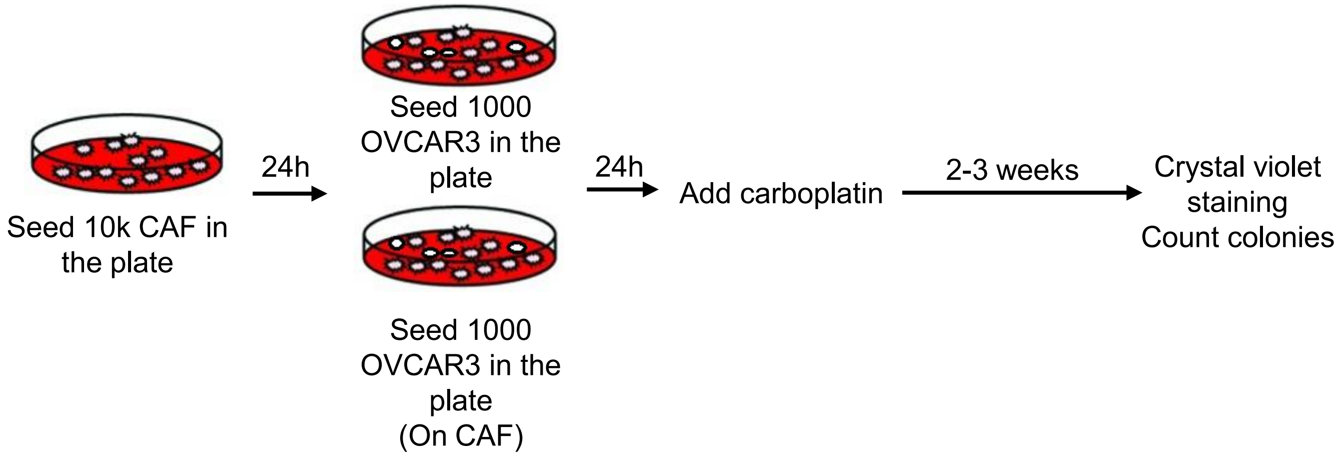

C

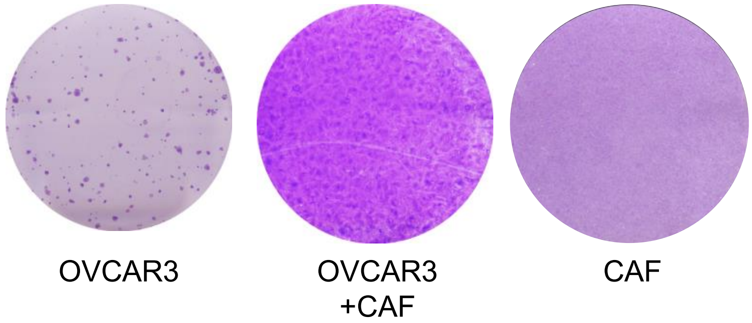

D

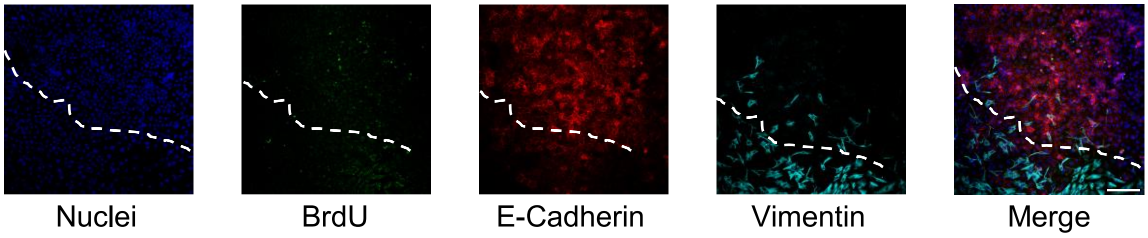

Supplementary Figure 3:

A: Marker expression in CAFs and OVCAR3 OC cells. OVCAR3 cells and CAFs were cultured on 10mm cover slip and fixed by 4% paraformaldehyde. Immunofluorescence staining was performed for pan-keratin (epithelial cell marker), vimentin and αSMA (CAF markers). Scale bar: 100μm.

B: Schematic outline of colony formation assay with/without CAFs for Figure 1D.

C: Representative images of colony formation assay (Figure 1D). Cells were stained with 0.01% crystal violet after 4% paraformaldehyde fixation. Colonies appear dark violet (Left and middle images) and CAFs do not form colonies and appear as a light violet monolayer (right image)

D: Interface interaction assay of OVCAR3 cells cocultured with CAFs. OVCAR3 cells and CAFs were seeded on 10mm coverslips separated by cloning ring. The ring was removed after 24h and cells were allowed to grow and merge at the interface followed by carboplatin treatment (33μM, IC_{50} using MTT assay). TUNEL assay was done to label apoptotic cells. Cancer cells and CAFs were stained with E-cadherin and Vimentin respectively. Images were obtained using Leica SP8 confocal microscope (10x objective). Scale bar: 200μm.

Supplementary Figure 4

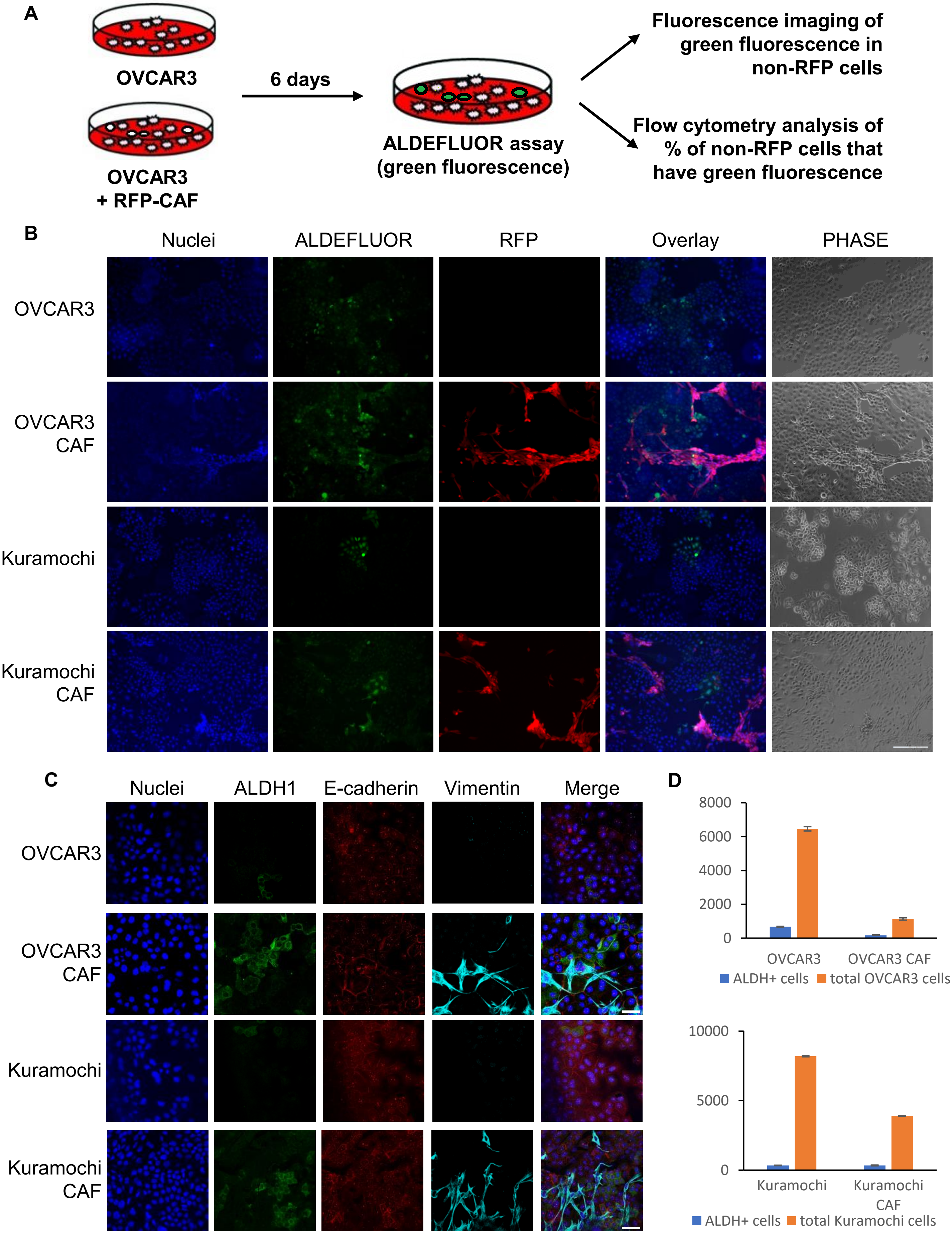

Supplementary Figure 4 (Continued)

E

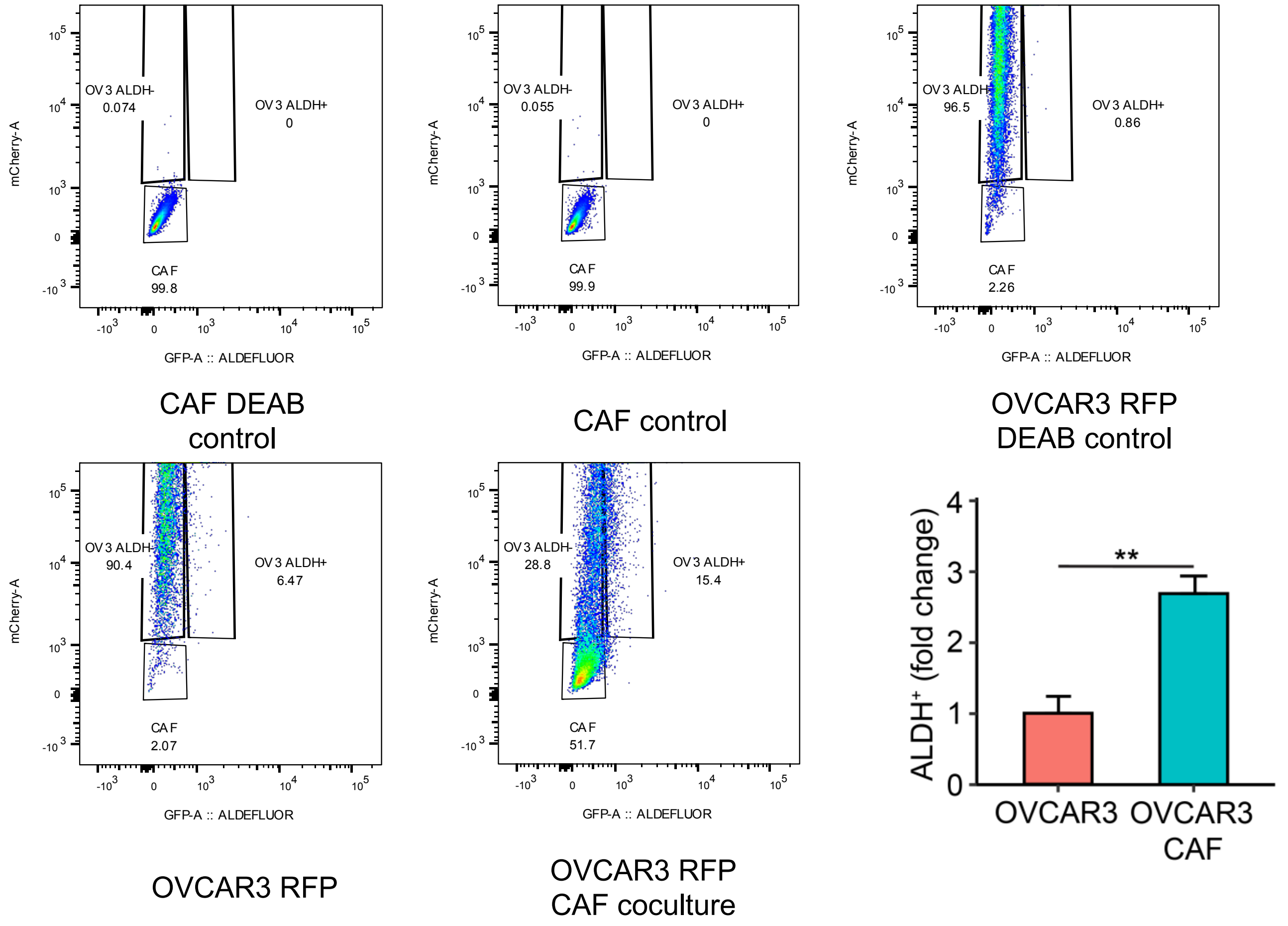

Supplementary Figure 4:

A: Schematic outline of OVCAR3-CAF coculture procedure. (For Figure 2A-B and subsequent assays)

B: ALDEFLUOR assay for stem cell enrichment in OC-CAF coculture with IWP2 treatment. OVCAR3/Kuramochi cells were seeded with CAFs and cocultured for a week with 5µM PORCN inhibitor IWP2. ALDEFLUOR assay was performed to label CSC (green). DAPI staining was used to label nuclei (blue). CAFs used had stable RFP expression to differentiate them from the OC cells in coculture. Scale bar: 100µm.

C: Immunofluorescence assay of OVCAR3/Kuramochi cells cocultured with CAFs. OVCAR3 cells and CAFs were seeded on 10mm and co-cultured for a week. CSCs were stained with ALDH1. Cancer cells and CAFs were stained with E-cadherin and Vimentin respectively. Images were obtained using a Leica SP8 confocal microscope (40x objective). Scale bar: 50µm.

D: Plots depicting the number of ALDH+ OVCAR3 (Top) or Kuramochi (Bottom) cells compared to the total number of respective OC cells in monoculture or coculture with CAF-RFP followed by ALDEFLUOR assay. Only the OC cells were counted, using flow cytometry, for each condition. Error bars depict Mean ± SD from 3 independent experiments.

E: ALDEFLUOR assay for stem cell enrichment in OC-CAF coculture. OVCAR3 cells were seeded with CAFs and cocultured for a week. ALDEFLUOR assay was performed to label CSC (green). OVCAR3 cells used had stable RFP expression to differentiate them from the CAFs in coculture. Flow cytometry analysis was done to quantify CSCs in control OVCAR3 cells and in OVCAR3 cocultured with CAFs. Mean ± SD from 3 independent experiments. ** $p<0.01$ (t-test)

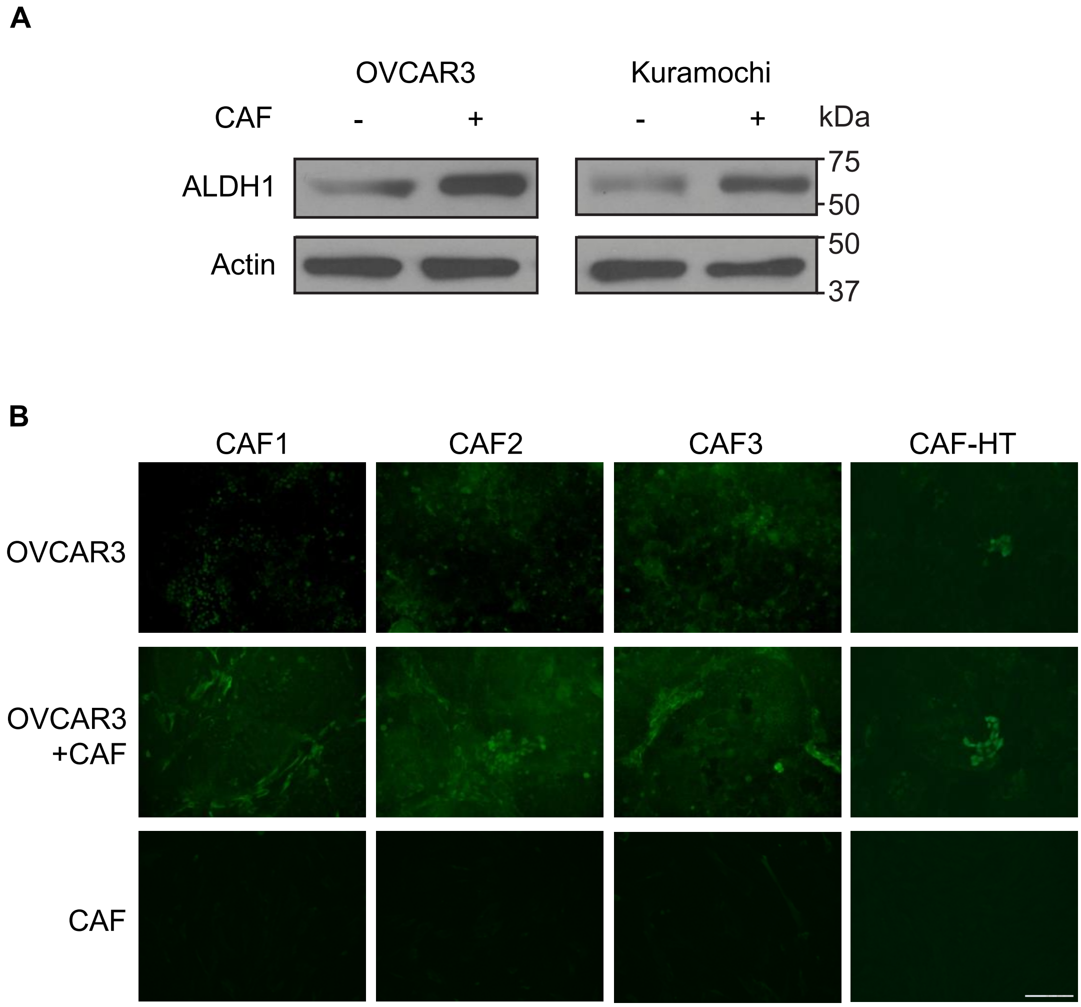

Supplementary Figure 5:

A: Representative immunoblots for OC cells cocultured with CAFs. OVCAR3/Kuramochi cells were cocultured with RFP expressing CAFs for 7 days, then separated by FACS and lysed with RIPA buffer. Proteins were separated using 4%-20% gradient SDS-PAGE and transferred to a nitrocellulose membrane. ALDH1 and actin were probed.

B: ALDEFLUOR assay for stem cell enrichment in OC-CAF coculture using CAFs isolated from OC patient tumors and grown as primary cultures for up to 5 passages. OVCAR3 cells were seeded with the primary CAFs and cocultured for a week. ALDEFLUOR assay was performed to label CSC (green fluorescence). Scale bar: 100μm.

Supplementary Figure 6

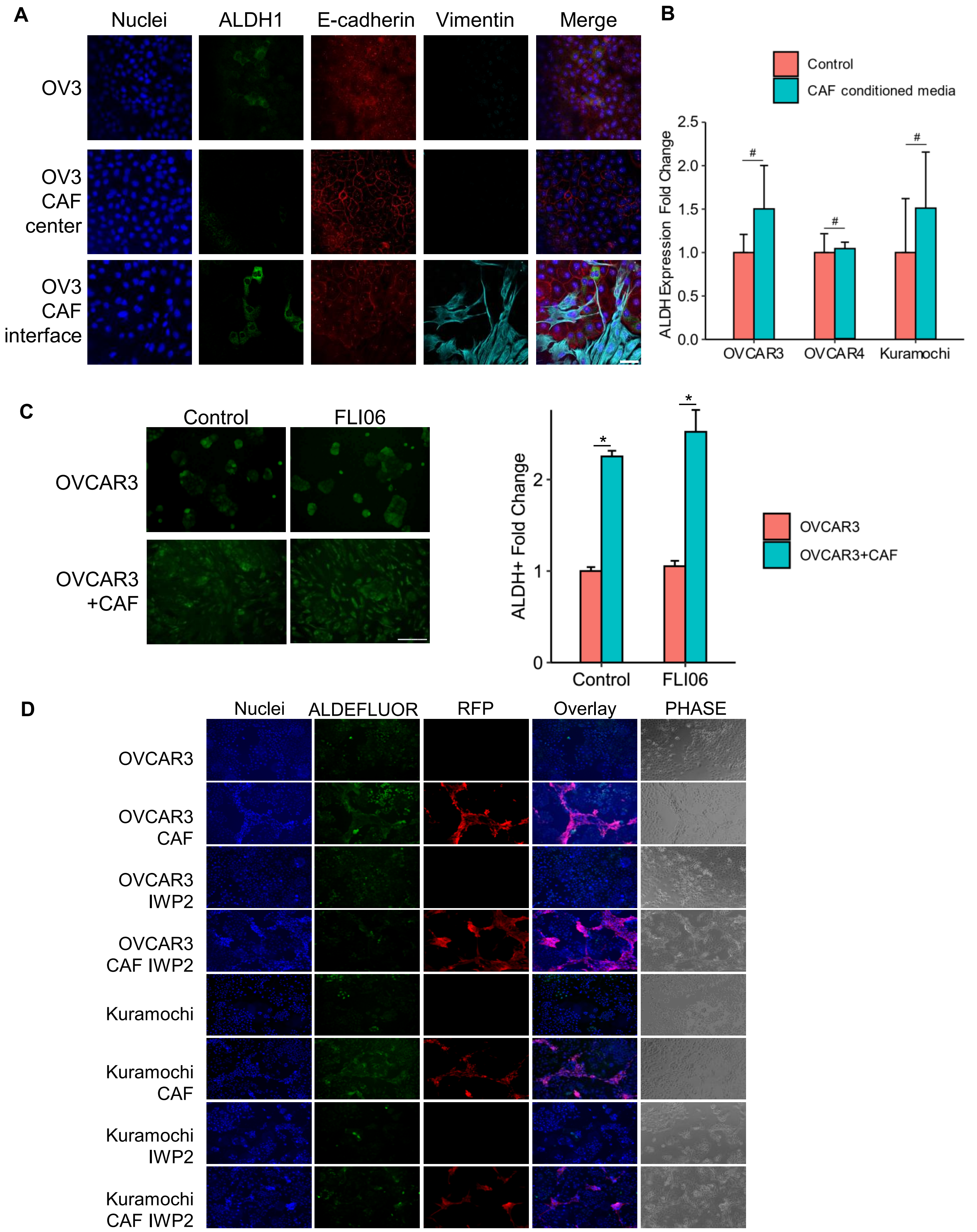

Supplementary Figure 6:

A: Interface interaction assay of OVCAR3 cells cocultured with CAFs. OVCAR3 cells and CAFs were seeded on 10mm coverslip separated by cloning ring. The ring was removed after 24h and cells were allowed to grow and merge at the interface. CSCs were stained with ALDH1. Cancer cells and CAFs were stained with E-cadherin and Vimentin respectively. Nuclei were stained with Hoechst 33342. Images were obtained using a Leica SP8 confocal microscope (40x objective). Scale bar: 50μm.

B: qPCR for ALDH1A1 expression in OC cells treated with CAF conditioned medium. Mean ± SD from 3 independent experiments. # not significant (t-test)

C: ALDEFLUOR assay for stem cell enrichment in OC-CAF coculture with Notch inhibitor (FLI06) treatment. OVCAR3 cells were seeded with CAFs and cocultured for a week and treated with increasing doses of FLI06 (showing 10nM, the maximum dose, without massive cell death). ALDEFLUOR assay was performed to label CSCs (green). Scale bar: 100μm. CSCs were quantified by ImageJ counting. Mean ± SD from 3 independent experiments. * $p<0.01$ (t-test)

D: ALDEFLUOR assay for stem cell enrichment in OC-CAF coculture treated with IWP2. OVCAR3/Kuramochi cells were seeded with RFP expressing CAFs and cocultured for a week with 5μM PORCN inhibitor IWP2. ALDEFLUOR assay was performed to label CSCs (green). Fluorescent imaging of OC-CAF coculture labeled by ALDEFLUOR. Nuclei were stained with Hoechst 33342. Scale bar: 100μm.

Supplementary Figure 7

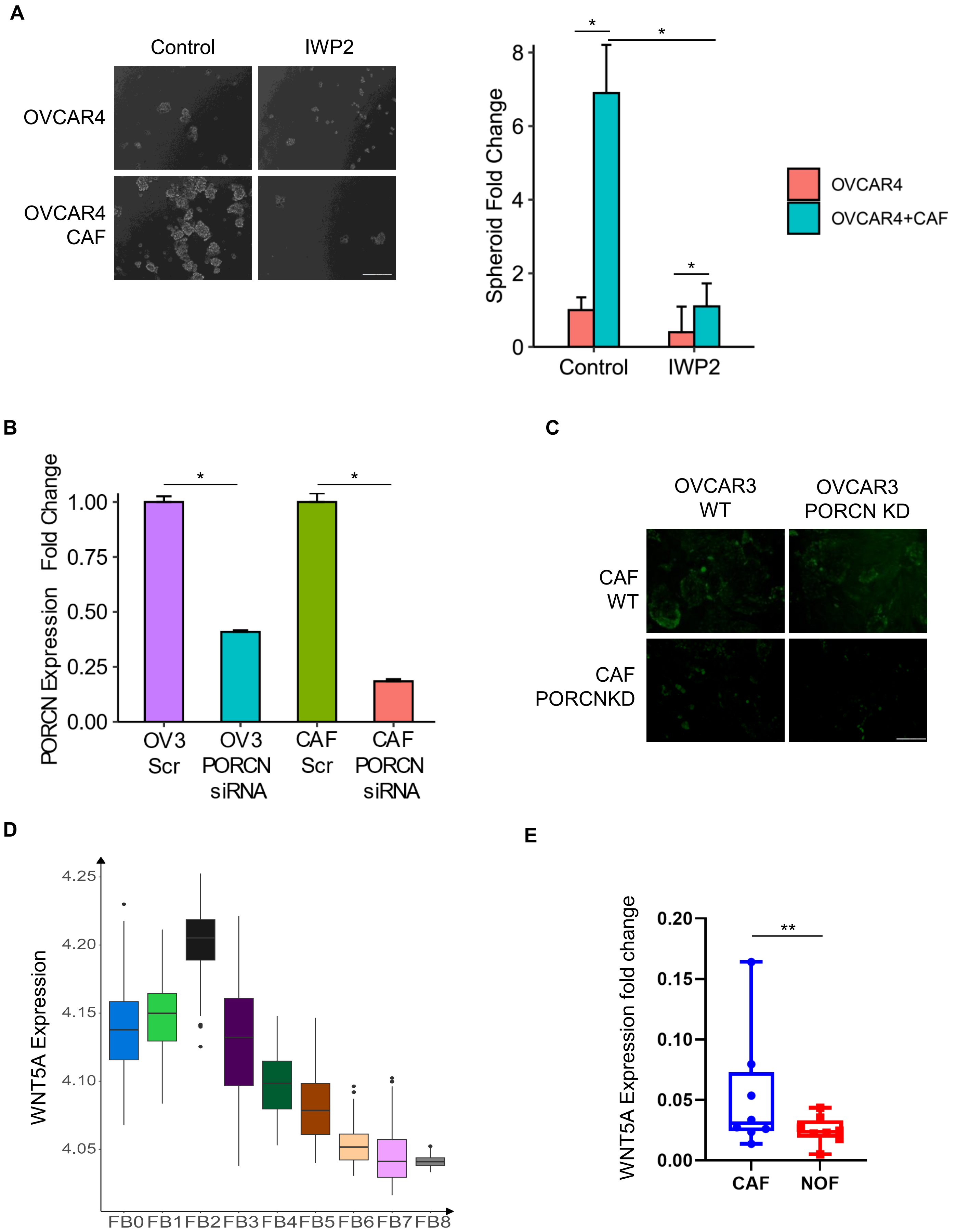

Supplementary Figure 7:

A: Spheroid formation assay of OC-CAF coculture. OVCAR4 cells were seeded with/without CAFs in ultra-low adhesion plate and cocultured for 14 days with/without 5μM PORCN inhibitor (IWP2). Images and quantification of number of spheroids are shown. Scale bar: 400μm. Mean ± SD from 3 independent experiments. * $p<0.01$ (t-test)

B: qPCR for PORCN silencing for experiments in Figure 3E. Mean ± SD from 3 independent experiments. * $p<0.01$ (t-test)

C: Knockdown of PORCN in OC/CAF: Scrambled negative control or PORCN siRNA was transfected in OVCAR3/CAF 48h before coculture as indicated. OVCAR3/CAF were then cocultured for a week. ALDEFLUOR assay was performed to label CSCs (green). Representative fluorescent images are shown. Scale bar: 100μm.

D: Boxplot of normalized expression level of WNT5A in different CAF subpopulations (FB0 - FB8) from the analysis of published scRNA-seq data of 11 HGSOC patients.

E: qPCR for Wnt5a expression levels in 7 different patient derived CAFs and 7 normal omental fibroblasts (NOFs). ** $p<0.05$ (t-test)

Supplementary Figure 8

A

Expression	WNT5A^{high}	WNT5A^{low}	P value
ALDH1A1	8.358198	6.914966	3.17E-08
NANOG	5.423602	5.220428	0.010421
SOX2	4.168246	4.384473	0.032899
PROM1	7.367459	6.428225	0.018941
KIT	6.720383	5.839716	3.33E-07
Average	6.407578	5.757562	1.66E-07

B

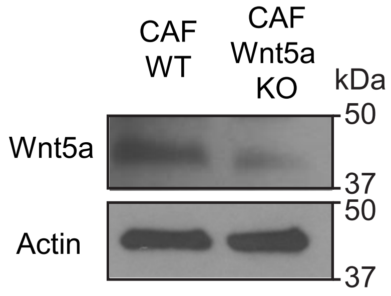

C

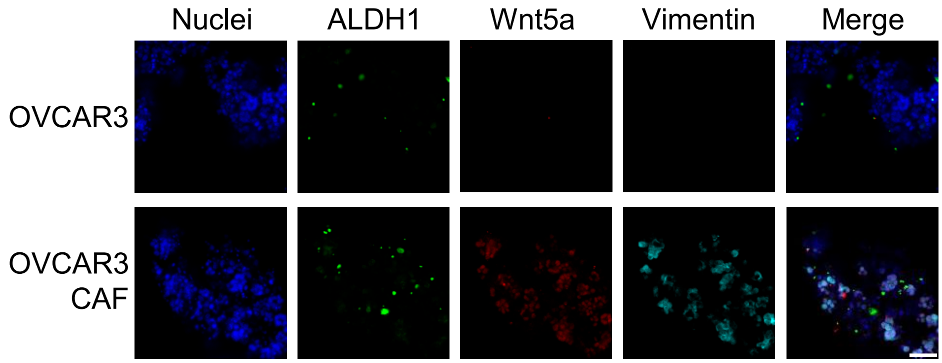

D

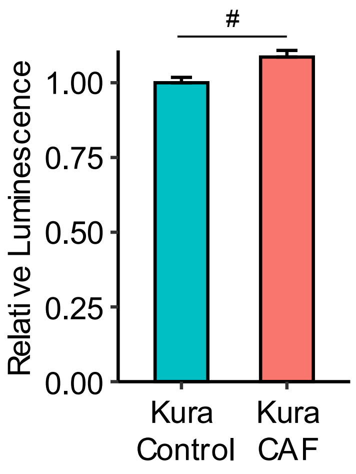

E

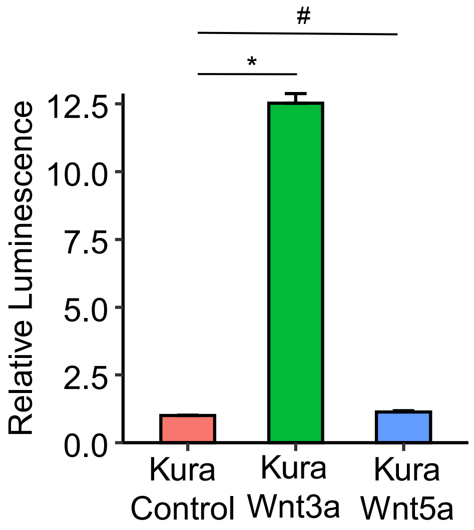

F

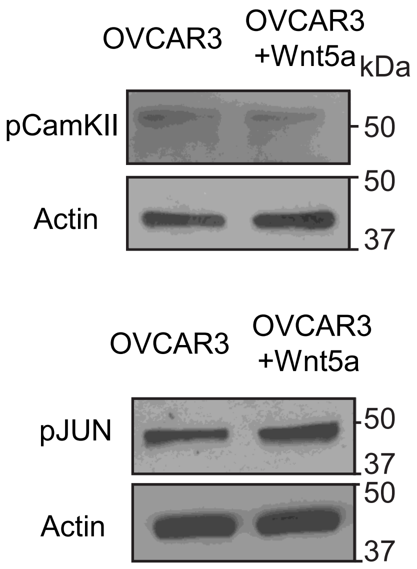

Supplementary Figure 8:

A: The oligo R package (version 1.54.1) was used to normalize the expression matrix from the AOCS dataset (285 patients). Average expression values of CSC markers (ALDH1A1, NANOG, SOX2, PROM1, KIT,) were compared in $WNT5A^{high}$ (top quartile) and $WNT5A^{low}$ (bottom quartile) patients.

B: Wnt5a expression in CAFs after CRISPR knockout was tested by immunoblotting. Cells were lysed with RIPA buffer. Proteins were prepared using 4%-20% gradient SDS-PAGE and transferred to a nitrocellulose membrane. Wnt5a and actin was probed. Since CAFs cannot form single colonies, clonal selection was not done to isolate Wnt5a-KO CAFs. So, this is a heterogenous population of CAFs, which include some wild type CAFs.

C: Immunofluorescent staining of heterotypic spheroids of OVCAR3+CAF or OVCAR3 monoculture. OVCAR3+CAF (1:1) were seeded in ultra-low adhesion plate for 7 days. The spheroids were isolated, fixed and stained for respective markers. ALDH1 was labeled green, Wnt5a was labeled cyan, and vimentin (CAF marker) was labeled red. Scale bar: 100µm.

D: Top/FOPFLASH assay showing TCF activity (downstream of canonical Wnt pathway) in Kuramochi cells cocultured with CAFs or monoculture control. A luciferase reporter (TCF promoter) was transfected into Kuramochi. 48h after transfection, the cells were cocultured with CAFs for 3 days and luciferase activity was measured. Mean ± SD from 3 independent experiments. # not significant (t-test)

E: Top/FOPFLASH assay showing TCF activity (downstream of canonical Wnt pathway) of Kuramochi cells treated with 200ng/mL recombinant human Wnt3a or Wnt5a. A luciferase reporter (TCF promoter) was transfected into Kuramochi. 48h later, the cells were treated with 200ng/mL recombinant human Wnt3a or Wnt5a for 3 days and luciferase activity was measured. Controls received PBS. Mean ± SD from 3 independent experiments. * $p<0.01$, # not significant (t-test)

F: Phosphorylated-CamKII and phosphorylated-Jun expression in OVCAR3 cells after Wnt5a treatment was tested by immunoblotting. OVCAR3 cells were seeded in 6-well plate, starved with serum-free DMEM for 24h, then treated with 200ng/mL Wnt5a for 2h followed by lysis and immunoblotting. Phosphorylated-CamKII, phosphorylated-Jun and actin was probed.

Supplementary Figure 9

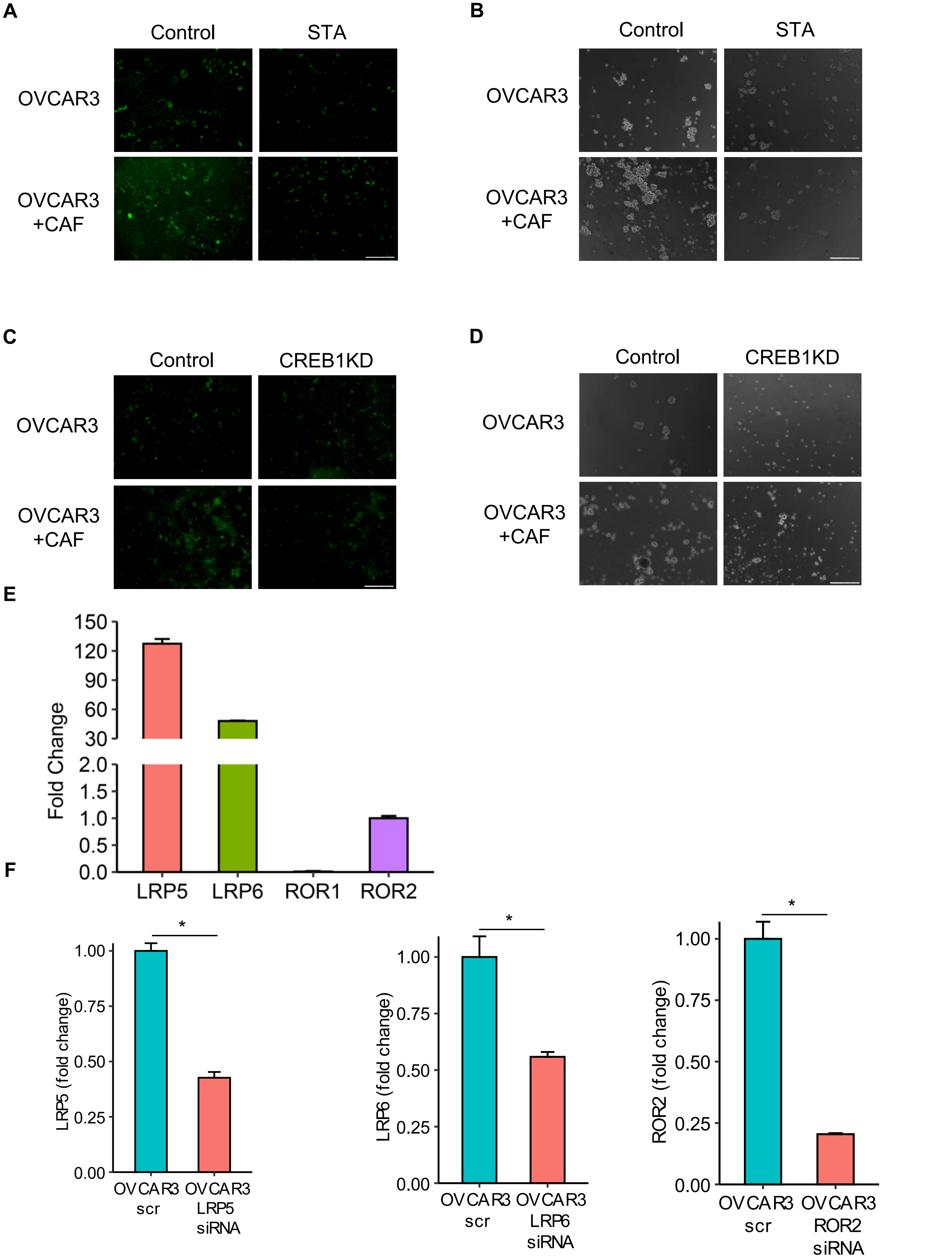

Supplementary Figure 9:

A: ALDEFLUOR assay for stem cell enrichment in OC-CAF cocultures with PKC inhibition. OVCAR3 cells were seeded with CAFs and cocultured for a week with 50nM PKC inhibitor Staurosporine (STA). ALDEFLUOR assay was performed to label CSCs (green). Fluorescent imaging of OC-CAF coculture labeled by ALDEFLUOR. Scale bar: 100μm.

B: Spheroid formation assay of OC-CAF coculture with Wnt5a inhibition. OVCAR3 cells were seeded with/without CAFs in ultra-low adhesion plates and cocultured for 14 days with 10nM PKC inhibitor Staurosporine (STA). Representative images are shown. Scale bar: 400μm.

C: : ALDEFLUOR assay for stem cell enrichment in OC-CAF cocultures with CREB1 silencing. OVCAR3 cells were transfected with CREB1 siRNA for 48h and then seeded with CAFs and cocultured for a week. ALDEFLUOR assay was performed to label CSCs (green). Fluorescent imaging of OC-CAF coculture labeled by ALDEFLUOR. Scale bar: 100μm.

D: : Spheroid formation assay of OC-CAF coculture with CREB1 silencing. OVCAR3 cells were transfected with CREB1 siRNA for 48h and then seeded with/without CAFs in ultra-low adhesion plates and cocultured for 14 days. Representative images are shown. Scale bar: 400μm.

E: Wnt5a co-receptor (LRP5, LRP6, ROR1 and ROR2) expression levels in OVCAR3 were measured by qPCR.

F: qPCR for LRP5, LRP6 and ROR2 silencing for experiments in Figure 6A-C. Mean ± SD from 3 independent experiments. * $p<0.01$ (t-test)

Supplementary Figure 10

A

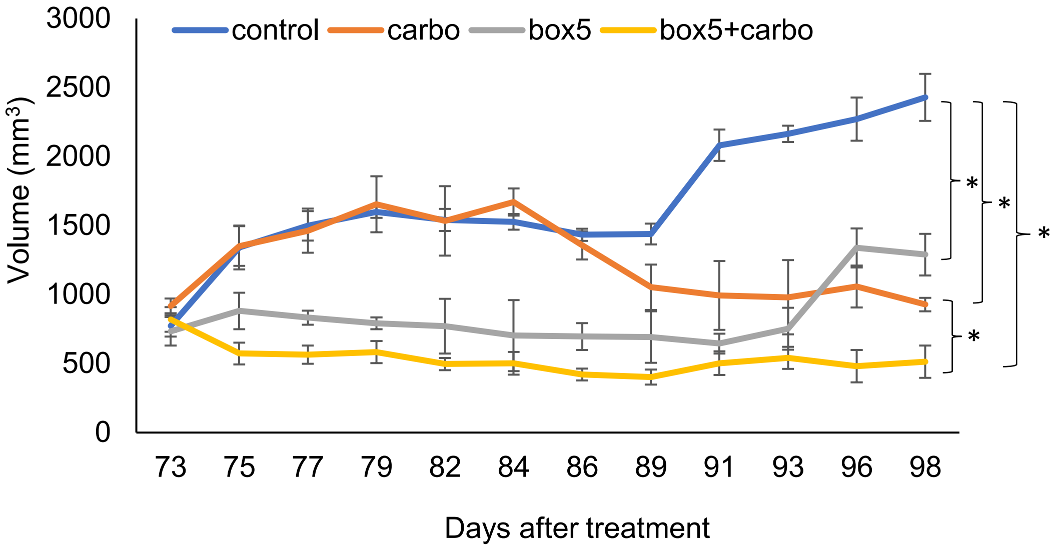

B

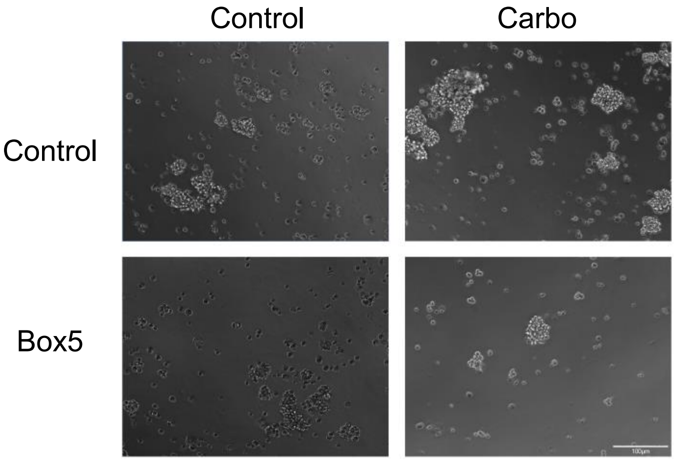

Supplementary Figure 10:

A: *In vivo* combination treatment of Box5/carboplatin in mouse xenografts. Mice were sub-cutaneously injected with 1 million OVCAR3+2million CAFs. When tumor size reached 1cm in diameter (on day70 of injection), mice were randomized into 4 groups of 5 mice per group and treated with 25mg/kg carboplatin weekly or 1.6mg/kg Box5, 3 times per week or a combination of both. The control group received PBS. Mice were euthanized after 4 weeks of treatment and tumor growth curves were plotted. 0.5 x diameter x radius^2 was used to estimate tumor size.

B: Residual tumors were dissociated using a gentleMACS dissociator and used for spheroid formation assay to measure the residual OCSC fraction. The spheroids were imaged and quantified. Scale bar: 400µm.

Supplementary Figure 11: FACS Analysis Data

Example of a FACS data analysis workflow:

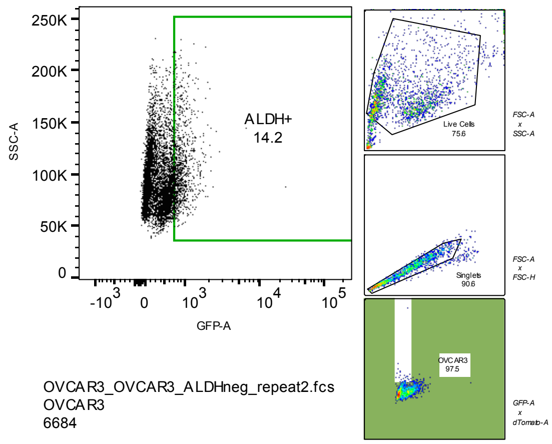

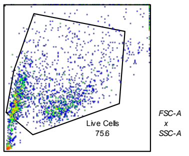

1. FSC-A and SSC-A gating for live cells

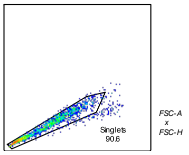

2. FSC-A and FSC-H gating for single cells

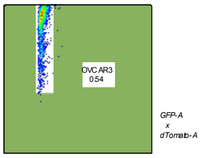

3. Run a pure CAF group, gate out CAFs (RFP positive)

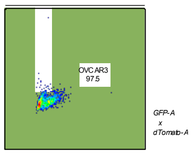

4. Run a pure OC cell group with that gate, adjust the gate to ensure OC cells in the gate (green)

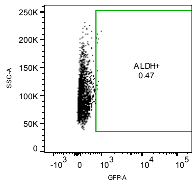

5. Run a DEAB group (ALDH inhibitor), make sure ALDH+ gate does not contain DEAB treated OC cells

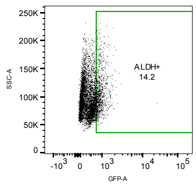

6. Run the actual experiment groups and quantify ALDH+ cells population towards all single OVCAR3 cells in the gate

FACS data for Figure 2

B

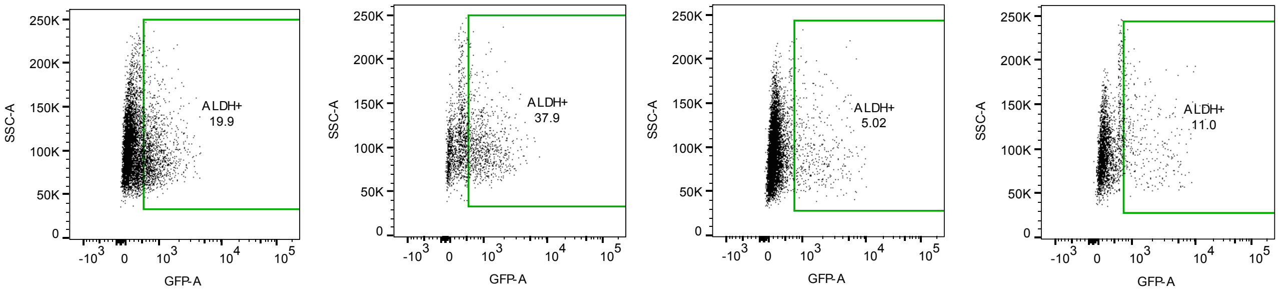

OVCAR3 | OVCAR3 +CAF | Kuramochi | Kuramochi +CAF

F

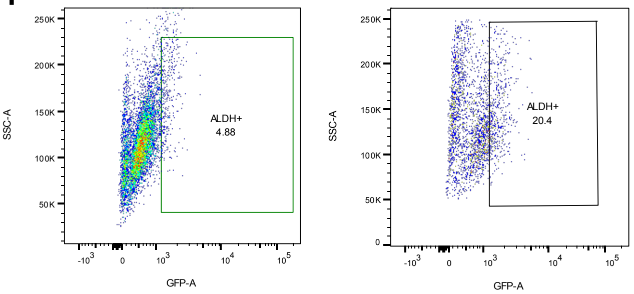

AS | AS +CAF

H

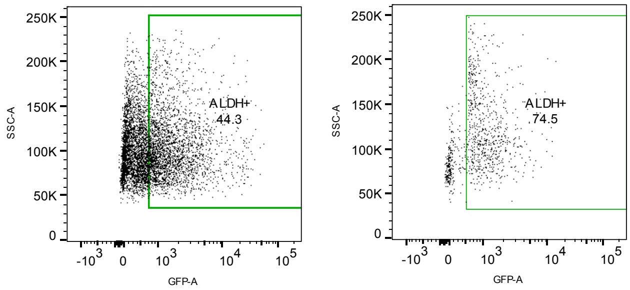

OVCAR3 | OVCAR3 +CAF

K

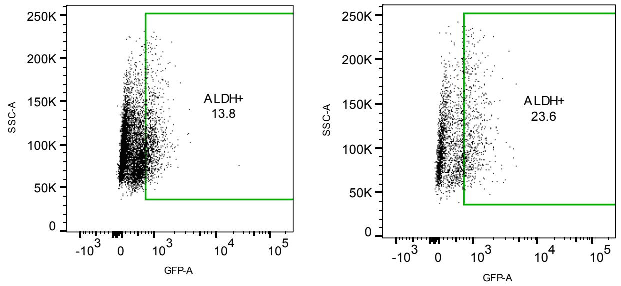

OVCAR3 | OVCAR3 +CAF

FACs data for Figure 3

B

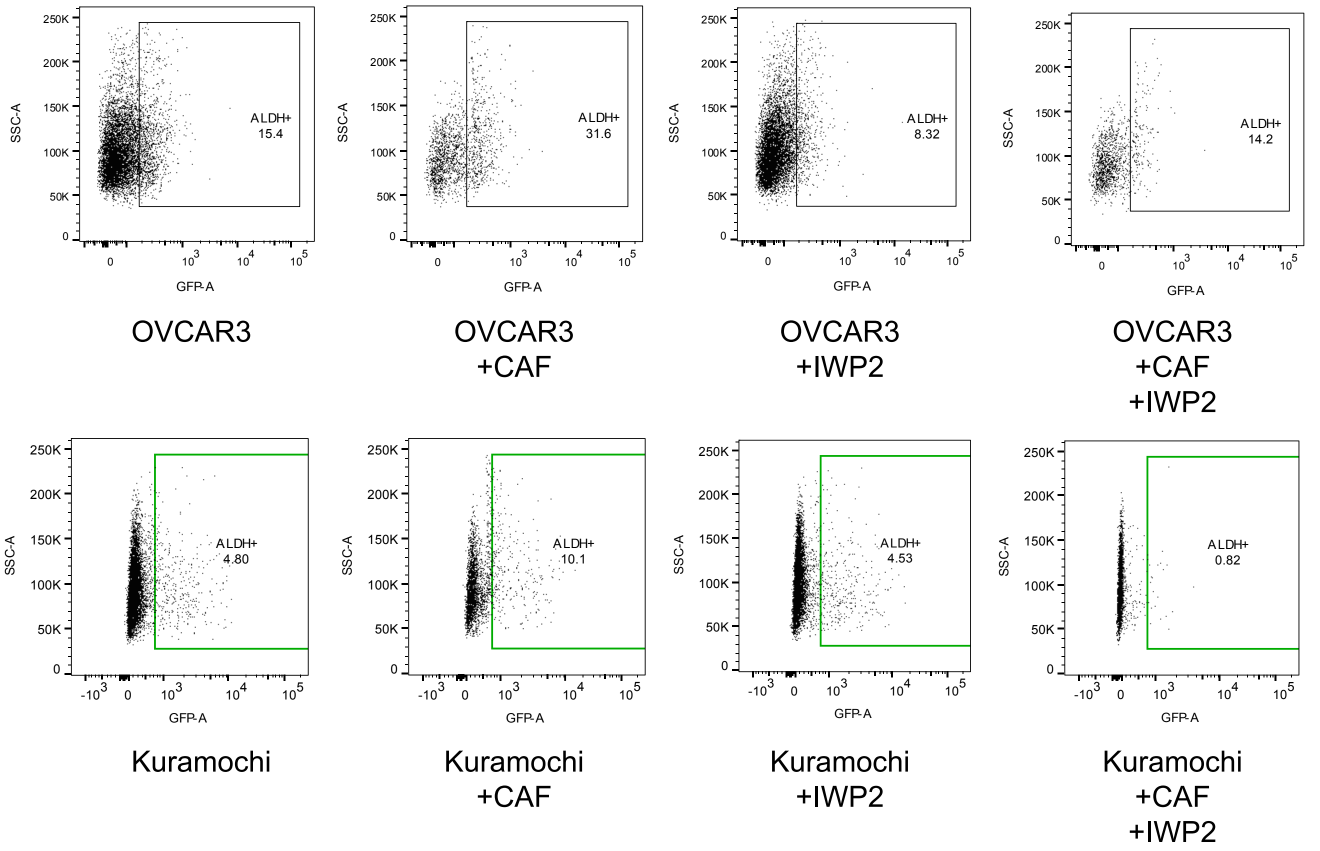

FACS data for Figure 4

A

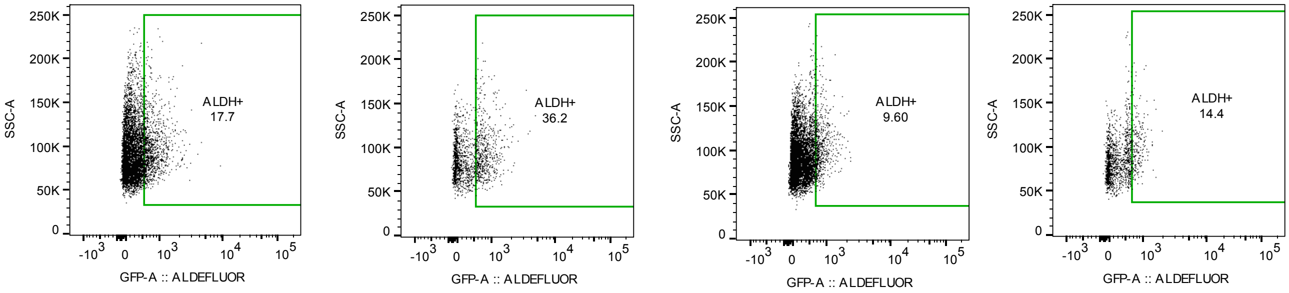

OVCAR3

OVCAR3
+CAF

OVCAR3
+Box5

OVCAR3
+CAF
+Box5

FACS data for Figure 5

E

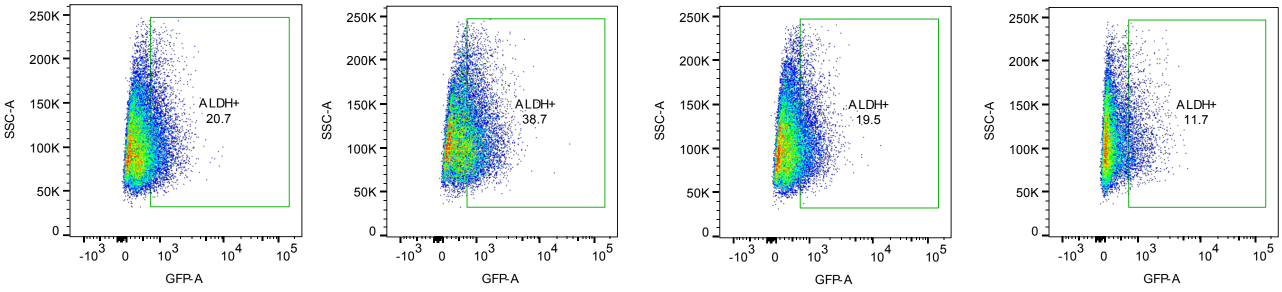

OVCAR3

OVCAR3
+CAF

OVCAR3
+Staurosporine

OVCAR3
+CAF
+Staurosporine

FACS data for Figure 5

J

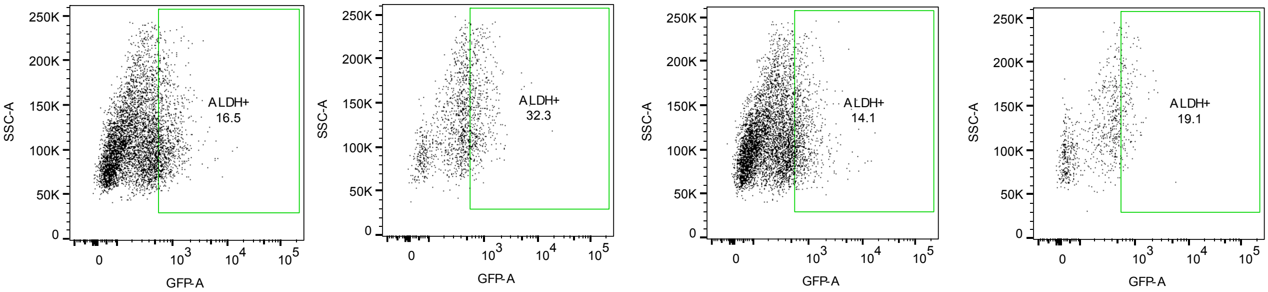

OVCAR3 | OVCAR3 +CAF | OVCAR3 CREB1KD | OVCAR3 CREB1KD +CAF

FACS data for Figure 6

B

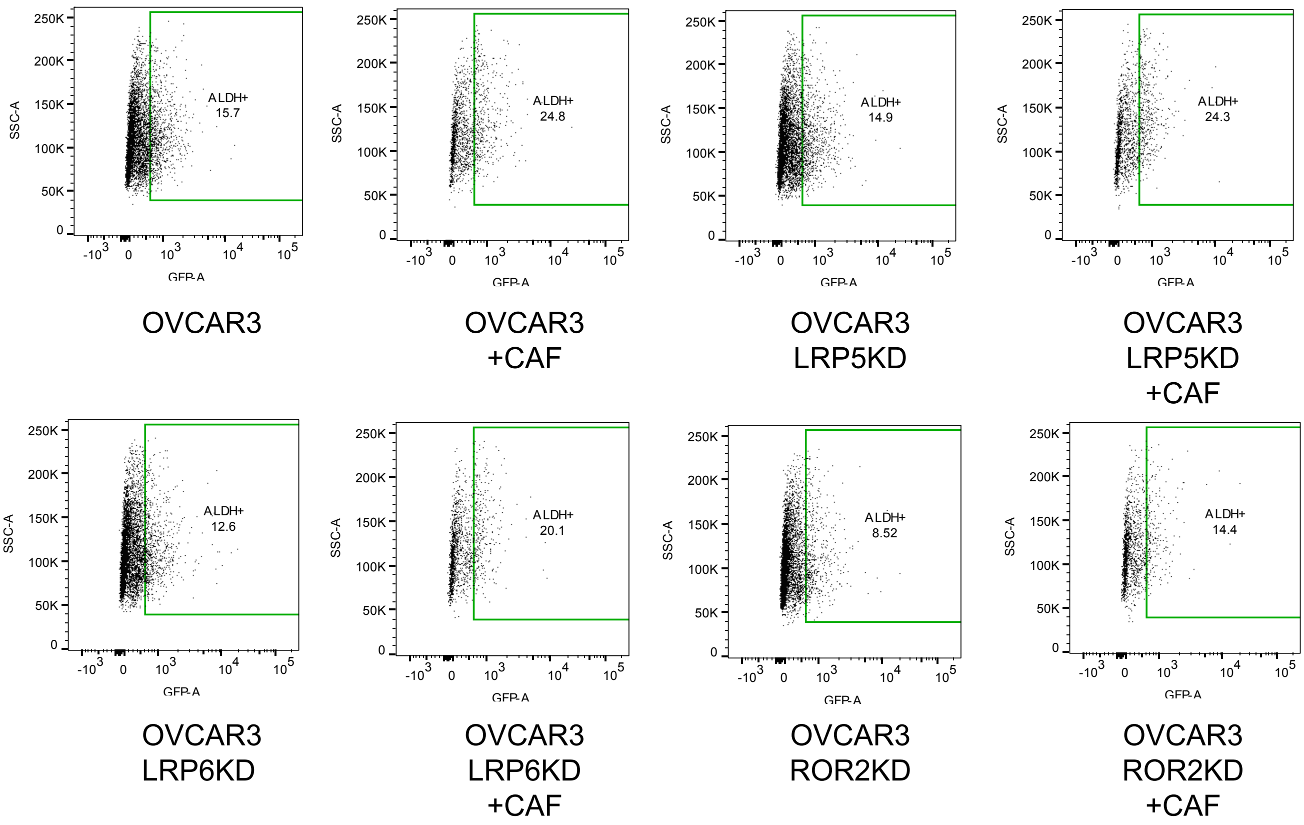

Supplementary Figure 12: Western Blots

Figure 3J Blots

Wnt5a

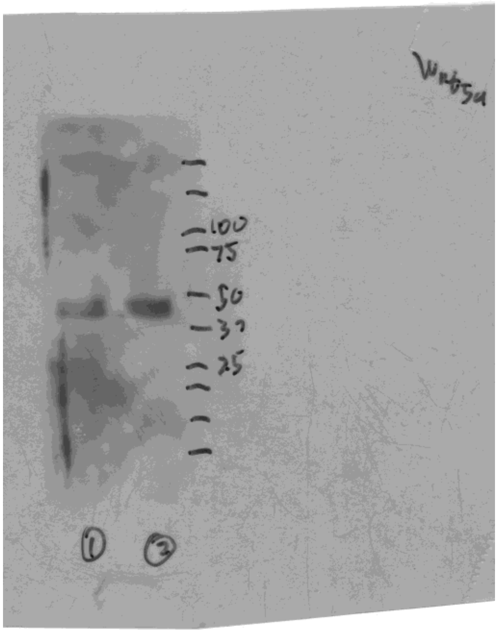

Actin

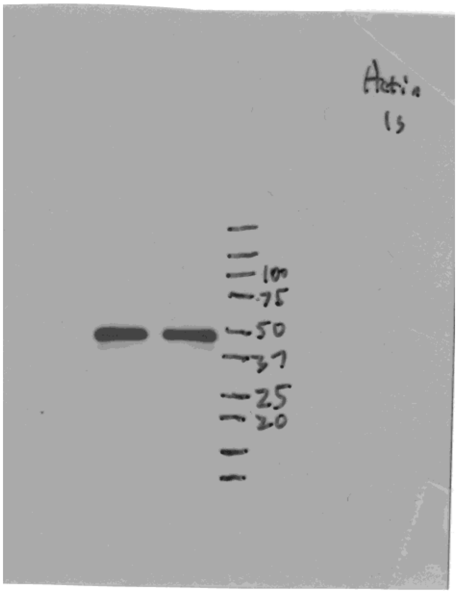

Figure 5B Blots

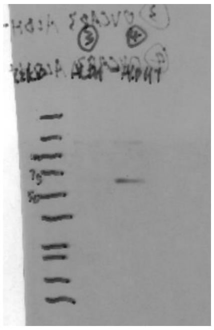

ALDH1

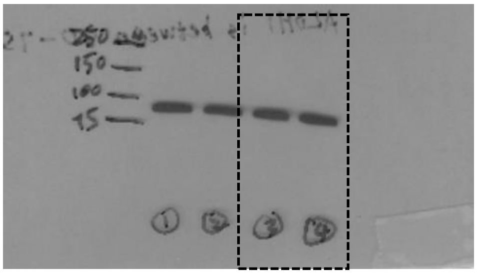

Unphospho Beta-catenin

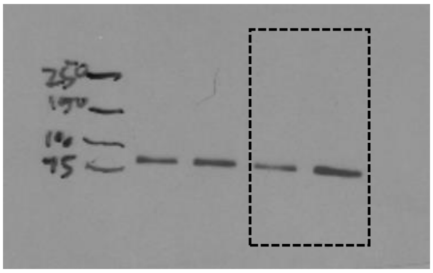

pPKC

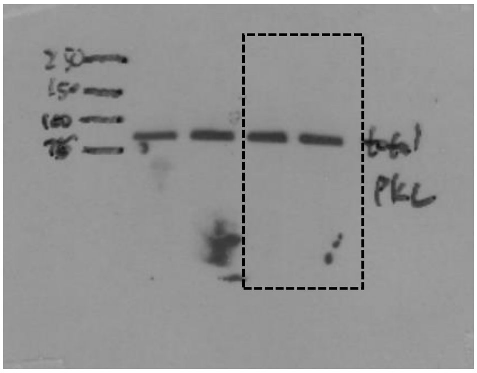

PKC

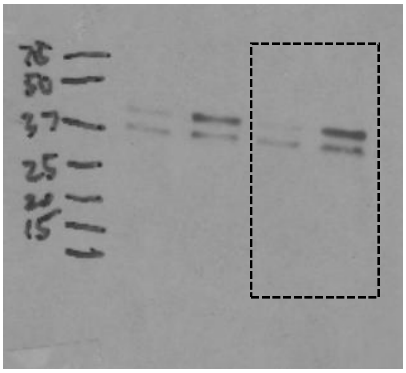

pCREB1

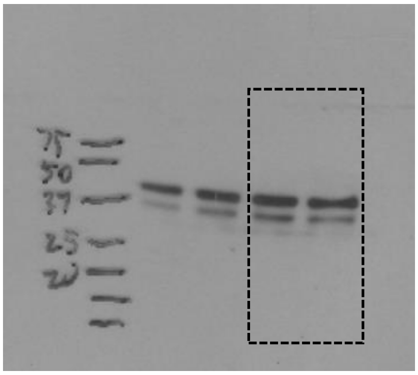

CREB1

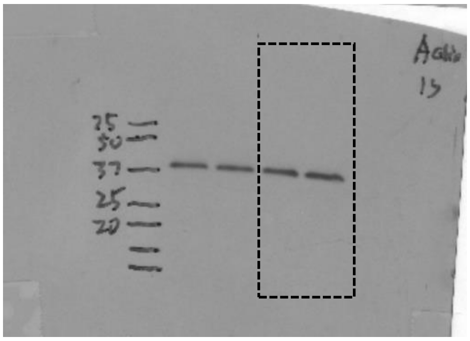

Actin

Figure 5C Blots

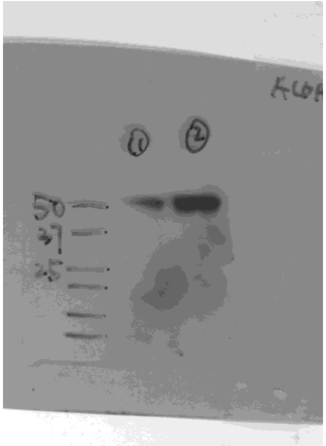

ALDH1

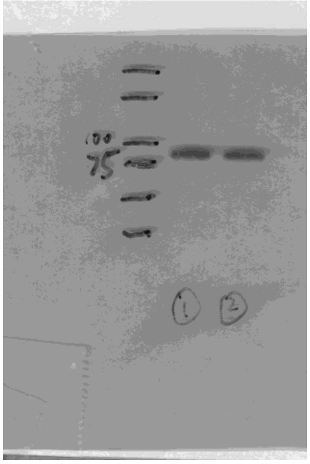

Unphospho
Beta-catenin

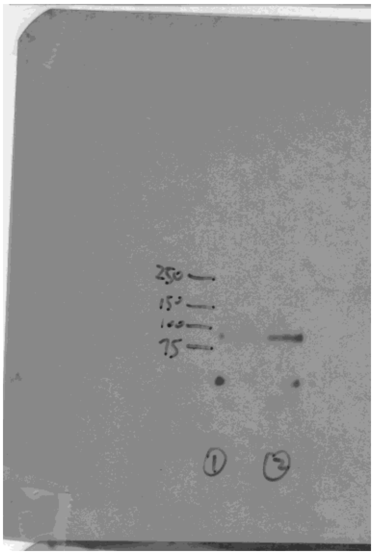

pPKC

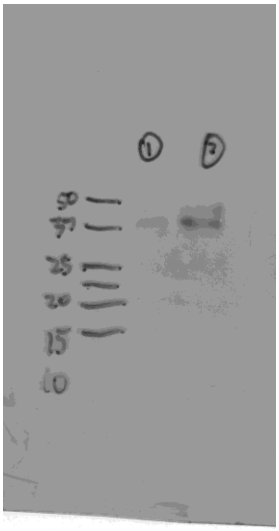

pCREB1

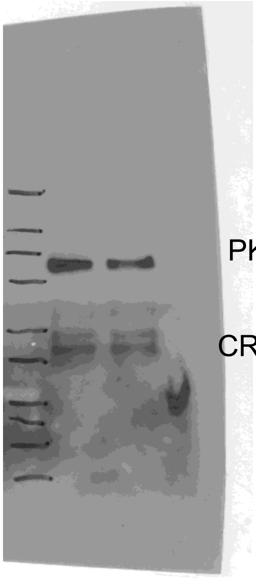

PKC

CREB1

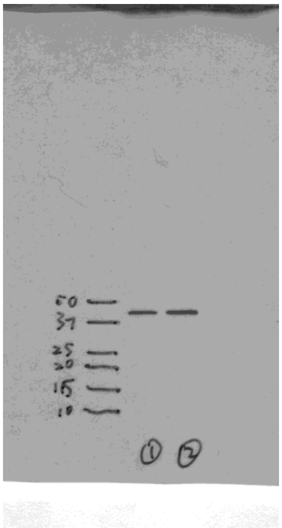

Actin

Figure 5D Blots

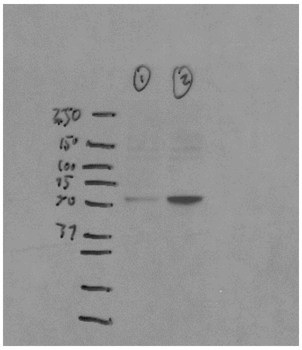

ALDH1

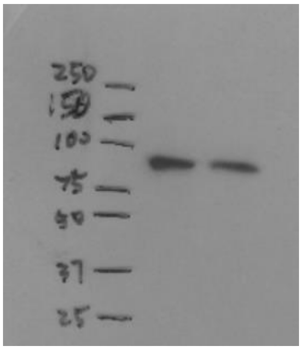

Unphospho
Beta-catenin

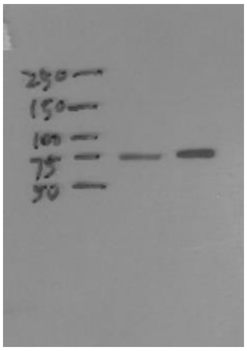

pPKC

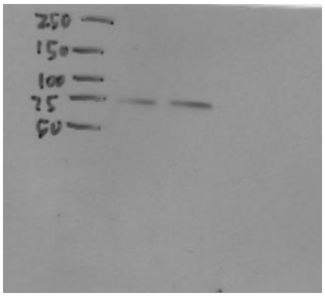

PKC

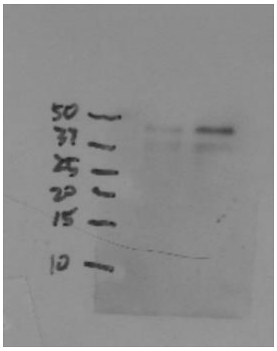

pCREB1

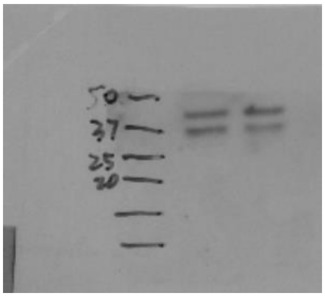

CREB1

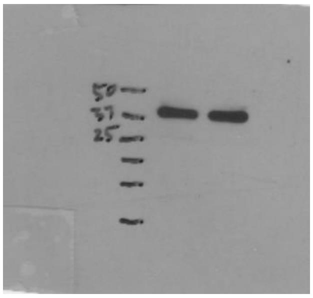

Actin

Figure 5G Blots

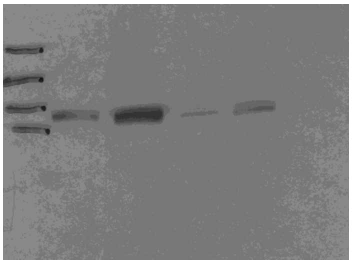

pPKC

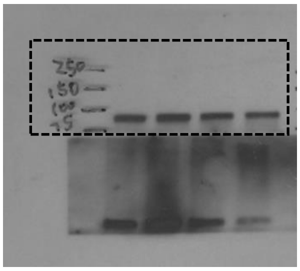

PKC

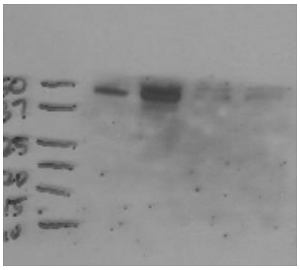

pCREB1

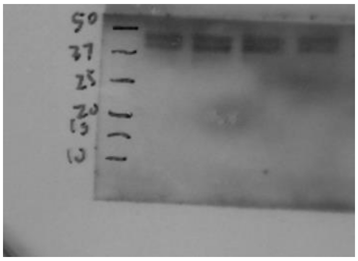

CREB1

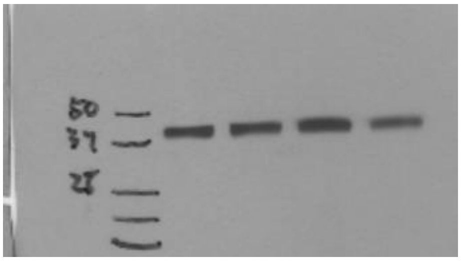

Actin

Figure 5H Blots

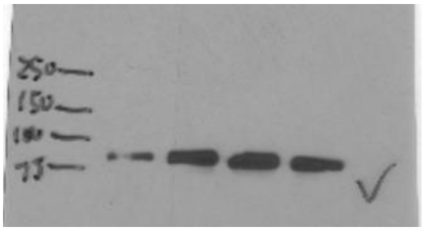

pPKC

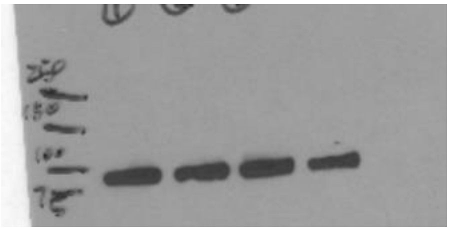

PKC

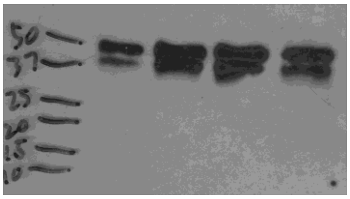

pCREB1

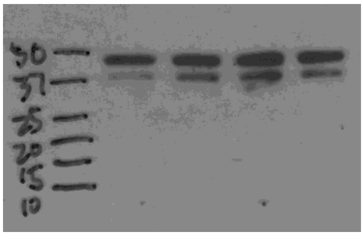

CREB1

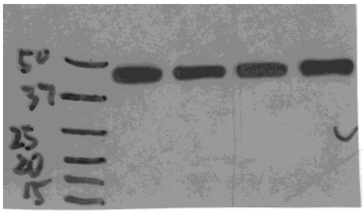

Actin

Figure 6F Blots

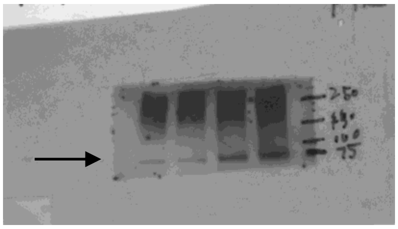

pPKC

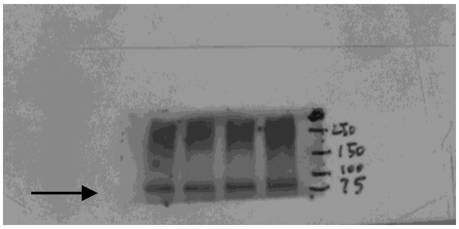

PKC

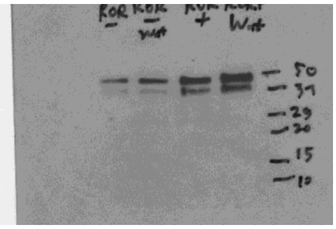

pCREB1

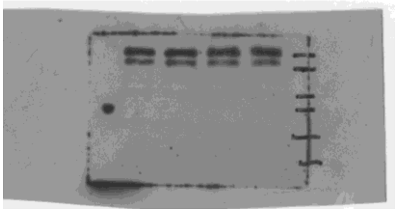

CREB1

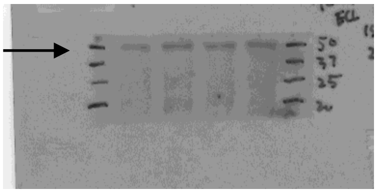

Actin
